# Definition and epidemiology of coronary microvascular disease

**DOI:** 10.1007/s12350-022-02974-x

**Published:** 2022-05-09

**Authors:** Conor Bradley, Colin Berry

**Affiliations:** 1 British Heart Foundation Glasgow Cardiovascular Research Centre, University of Glasgow, Glasgow, United Kingdom; 2 NHS Golden Jubilee Hospital, Clydebank, United Kingdom; 3 British Heart Foundation Glasgow Cardiovascular Research Centre, Institute of Cardiovascular and Medical Sciences, University of Glasgow, 126 University Place, Glasgow, G12 8TA Scotland United Kingdom

**Keywords:** Ischemic heart disease, microvascular angina, imaging, ischemia

## Abstract

Ischemic heart disease remains one of the leading causes of death and disability worldwide. However, most patients referred for a noninvasive computed tomography coronary angiogram (CTA) or invasive coronary angiogram for the investigation of angina do not have obstructive coronary artery disease (CAD). Approximately two in five referred patients have coronary microvascular disease (CMD) as a primary diagnosis and, in addition, CMD also associates with CAD and myocardial disease (dual pathology). CMD underpins excess morbidity, impaired quality of life, significant health resource utilization, and adverse cardiovascular events. However, CMD often passes undiagnosed and the onward management of these patients is uncertain and heterogeneous. International standardized diagnostic criteria allow for the accurate diagnosis of CMD, ensuring an often overlooked patient population can be diagnosed and stratified for targeted medical therapy. Key to this is assessing coronary microvascular function—including coronary flow reserve, coronary microvascular resistance, and coronary microvascular spasm. This can be done by invasive methods (intracoronary temperature-pressure wire, intracoronary Doppler flow-pressure wire, intracoronary provocation testing) and non-invasive methods [positron emission tomography (PET), cardiac magnetic resonance imaging (CMR), transthoracic Doppler echocardiography (TTDE), cardiac computed tomography (CT)]. Coronary CTA is insensitive for CMD. Functional coronary angiography represents the combination of CAD imaging and invasive diagnostic procedures.

**Supplementary Information:**

The online version contains supplementary material available at 10.1007/s12350-022-02974-x.

## Introduction

Ischemic heart disease (IHD) remains one of the leading causes of death and disability worldwide.^1,2^ Angina is the most common symptom of IHD, and the investigation and management of angina is often focused on the detection and treatment of flow-limiting epicardial coronary artery disease (CAD).^3^ However, most patients referred for an invasive coronary angiogram for the investigation of stable anginal symptoms do not have obstructive CAD.^4^ Multiple studies have shown that a large proportion of these patients have coronary microvascular dysfunction (CMD).^5–7^ However, this condition is rarely diagnosed and the onward management of these patients is uncertain and heterogenous. CMD is not benign. It is associated with increased risk of major adverse cardiovascular events,^8,9^ persistent anginal symptoms,^10^ impaired quality of life,^11^ and considerable health resource utilization due to recurrent hospitalizations and repeat invasive angiograms.^8^ Recent studies have highlighted the importance of establishing an accurate diagnosis and have demonstrated that stratified medical therapy leads to a marked and sustained improvement in angina symptoms and quality of life.^5,12^

## Definition

Camici and Crea defined Type 1 CMD as coronary microvascular dysfunction in the absence of obstructive CAD and myocardial diseases (Table 1).^6^ Type 2 CMD associates with cardiomyopathy, Type 3 CMD occurs in the presence of obstructive CAD, and Type 4 CMD is iatrogenic. Information gaps on the definition of microvascular angina and vasospastic angina stimulated the Coronary Vasomotion Disorders International Study Group (COVADIS), to set out standardized diagnostic criteria for the for the diagnosis of these conditions.^13^ The criteria for diagnosis required: (1) Symptoms of myocardial ischemia; (2) Absence of obstructive CAD; (3) Objective evidence of myocardial ischemia; (4) Evidence of impaired coronary microvascular function (Table 2). They define “Definite MVA” as the presence of all four criteria. However, “Suspected MVA” is diagnosed if criteria (1) and (2) are present, in addition to either (3) or (4). Therefore, a cornerstone of making the diagnosis of CMD is the use of diagnostic tests to specifically assess coronary microvascular function.

**Table 1:** Clinical classification of coronary microvascular dysfunction

Type	Clinical setting
1 In the absence of obstructive CAD or myocardial disease	Risk factors Microvascular angina
2 In the presence of myocardial diseases	Hypertrophic cardiomyopathy Dilated cardiomyopathy Anderson-Fabry’s disease Amyloidosis Myocarditis Aortic stenosis
3 In the presence of obstructive CAD	Stable angina Acute coronary syndrome
4 Iatrogenic	Percutaneous coronary intervention Coronary artery grafting

**Table 2:** COVADIS clinical criteria for suspecting microvascular angina

(1) Symptoms of myocardial ischemia (a) Effort and/or rest angina (b) Angina equivalents (i.e., shortness of breath)
(2) Absence of obstructive CAD (< 50% diameter reduction of FFR > 0.80) by (a) Coronary CTA (b) Invasive coronary angiogram
(3) Objective evidence of myocardial ischemia (a) Ischemic ECG changes during an episode of chest pain (b) Stress-induced chest pain and/or ischemic ECG changes in the presence or absence of transient/reversible abnormal myocardial perfusion and/or wall motion abnormality
(4) Evidence of impaired coronary microvascular function (a) Impaired coronary flow reserve (cut-off values depending on methodology use between < 2.0 and < 2.5) (b) Coronary microvascular spasm, defined as reproduction of symptoms, ischemic ECG shifts but no epicardial spasm during acetylcholine testing (c) Abnormal coronary microvascular resistance indices (e.g., IMR > 25) (d) Coronary slow-flow phenomenon, defined as TIMI frame count > 25

Definitive MVA is only diagnosed if all four criteria are present for a diagnosis of micro-vascular angina.

Suspected MVA is diagnosed if symptoms of ischemia are present (criteria-1) with no obstructive coronary artery disease (criteria-2) but only (a) objective evidence of myocardial ischemia (criteria-3), or (b) evidence of impaired coronary microvascular function (criteria-4) alone.^13^

*ECG*, electrocardiogram; *CAD*, coronary artery disease; *CTA*, computed tomographic angiography; *FFR*, fractional flow reserve; *IMR*, index of microcirculatory resistance; *TIMI*, thrombolysis in myocardial infarction

## Assessment of Coronary Microvascular Function

**Figure 1:**
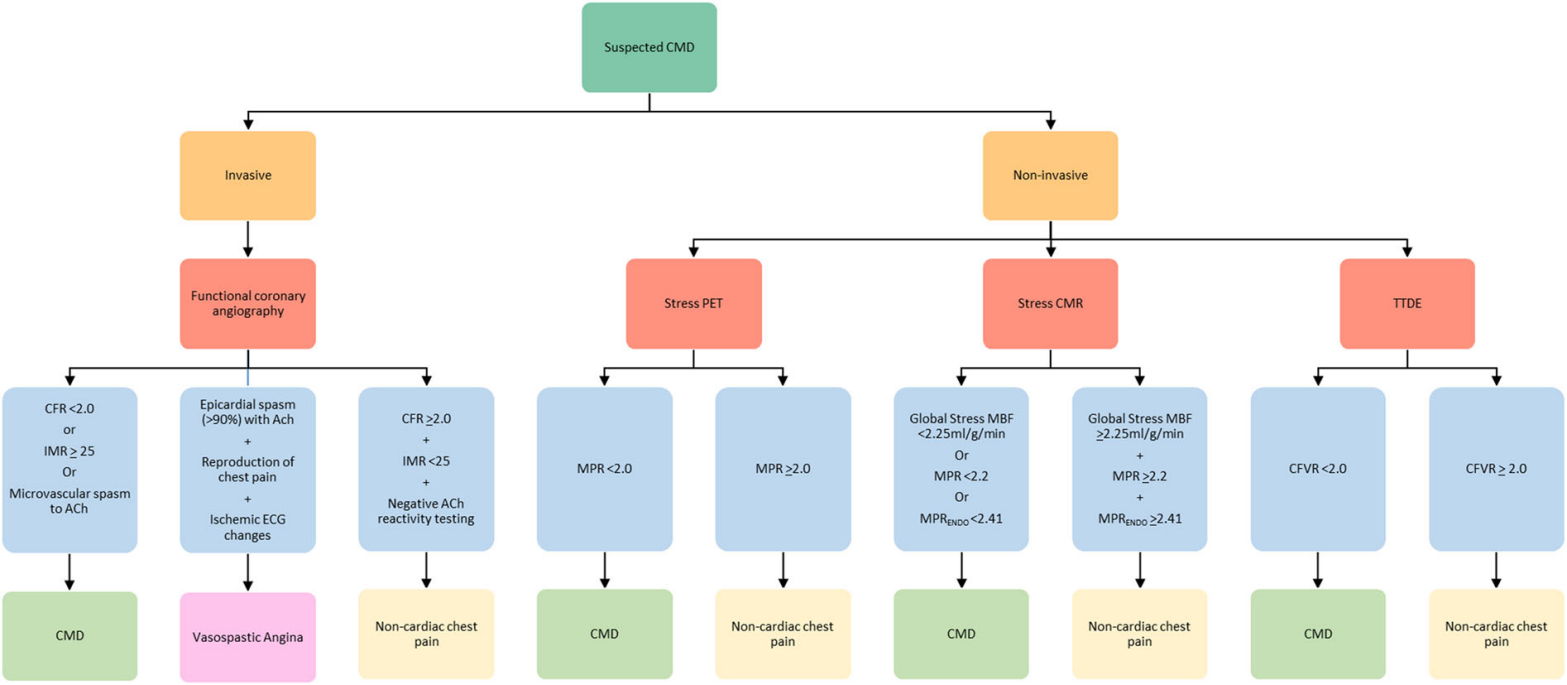
Algorithm for the investigation of patients with suspected CMD

Options for CMD evaluation include invasive and non-invasive techniques, each with strengths and limitations. Contemporary guidelines recommend that the test choice should be guided by local availability and expertise.^14^ A detailed description of each technique used to diagnose CMD will be covered in other manuscripts in this themed issue. These techniques are summarized below along with a proposed algorithm for the investigation of patients with suspected CMD (Figure 1).

### Invasive Techniques

The combination of coronary angiography with adjunctive tests of coronary vascular function is described as ‘functional coronary angiography’. A key attribute of invasive assessment of coronary microvascular function is the ability to test both endothelium-dependent and -independent pathways, which is not yet possible with non-invasive methods. Novel approaches, such as the interventional diagnostic procedure (IDP),^15^ combine direct measurements of microvascular function using guidewire sensors with pharmacological coronary reactivity testing, such as with acetylcholine (ACh).

The 2019 ESC CCS guidelines^16^ and the 2021 ACC/AHA Guideline for the Evaluation and Diagnosis of Chest pain^14^ have both given a IIa recommendation (‘should be considered’) to the use of guidewire-based measurement of coronary flow reserve (CFR) and/or measurements of microvascular resistance in patients with non-obstructive CAD and persistent chest pain symptoms. Similarly, intracoronary ACh testing can be used to assess for endothelium-dependent coronary microvascular spasm, supported by a IIa recommendation in ACC/AHA guidelines,^14^ and IIb in ESC guidelines.^16^ These trial recommendations are underpinned by the results of the coronary microvascular angina (CorMicA) randomized, controlled trial of stratified medical therapy.^5,12^

### Coronary Flow Reserve

CFR reflects the vasodilatory capacity of the coronary microcirculation. It is assessed by calculating the ratio of maximal hyperemic blood flow (usually induced by the intravenous infusion of a vasodilator such as adenosine), to resting blood flow. CFR gives a measure of flow through both the epicardial coronary arteries and the coronary microcirculation. However, in the absence of obstructive epicardial disease, CFR can be used as a marker of CMD.

CFR can be measured by a thermodilution technique using a pressure-temperature sensor guidewire, which estimates CFR indirectly using a saline bolus, by dividing resting mean transit time by hyperemic mean transit time. CFR can also be measured using a Doppler flow wire represented by flow velocity divided by resting flow velocity. In the absence of obstructive CAD, a CFR > 2.5 is considered normal, and a CFR < 2.0 is abnormal.^6,13,17^ An impaired CFR underpins a functional vasomotor disorder, i.e., functional microvascular angina.

## Measures of Microvascular Resistance

CMD may also be caused by an increase microvascular resistance, which underpins a structural vasomotor disorder, i.e., structural microvascular angina. Microvascular resistance can be measured in one or more coronary artery territories using a guidewire sensor and coronary thermodilution,^18^ including by use of intravenous adenosine^18^ or continuous intracoronary infusion of saline pressure and flow.^19^ This can be measured by combining pressure and flow measurements. Unlike CFR, this is independent of epicardial vascular function and is specific to the microcirculation. An additional benefit is that measures of microvascular resistance are independent of resting coronary flow.

The index of microvascular resistance (IMR) is measured using a manual saline bolus thermodilution technique and is calculated as the product of distal coronary pressure at maximal hyperemia multiplied by the hyperemic mean transit time.^18^ An IMR > 25 is abnormal, and consistent with a diagnosis of CMD.^20^ Alternatively, hyperemic myocardial velocity resistance (HMR) can be measured using a Doppler-based technique. This is calculated by dividing intracoronary pressure by hyperemic flow velocity. Studies have suggested a HMR >2.5 mmHg·cm^−1^·s^−1^ is consistent with CMD,^21^ however, there is less consensus around this cut-off and further validation is required.

The coronary resistive reserve ratio (RRR, derived using saline bolus thermodilution)^22^ and relatedly, myocardial resistance reserve (MRR, derived using continuous thermodilution),^23^ reflect microvascular resistance. MRR is derived from CFR divided by fractional flow reserve (FFR) corrected for driving pressure.^23^

## TIMI Frame Count

TIMI frame count provides a semi-quantitative method of assessing microvascular resistance. In the absence of obstructive CAD, a corrected TIMI frame count > 27 (images acquired at 30 frame·s^−1^)^24^ suggests MVA due to impaired resting flow (coronary slow-flow phenomenon).^13^ While this technique is low cost and easy to perform, it is less sensitive than other invasive techniques.

## Pharmacological Intracoronary Reactivity Testing

Coronary vasomotor tone is a function of endothelium-mediated vasorelaxation and constriction mediated by the vascular smooth muscle cells (VSMC). The vasomotor effects of vasoactive substances is a function of endothelial and non-endothelial responses. Vasoactive substances that mainly exert effects on the endothelium include ACh, ergonovine, and substance P.^24^ However, their effects are dose-dependent. ACh is the most widely used in clinical practice and can be used to assess endothelial function (low dose, < 1 µg dose of ACh), microvascular spasm (1-50 µg dose of ACh), (which causes MVA), as well as epicardial vasospasm (which causes vasospastic angina) (> 50-150 µg dose of ACh). The pharmacological protocols vary somewhat between practitioners but the general objectives are the same. This is achieved by briefly infusing ACh at increasing doses and assessing response. In healthy individuals, ACh stimulates nitric oxide release from the endothelium, leading to vasodilation of vascular smooth muscle. However, in patients with MVA it causes coronary microvascular spasm, defined as reproduction of symptoms, ischemic ECG shift, but no angiographic evidence of epicardial spasm.^13^

## Non-invasive Techniques

There are multiple non-invasive methods for assessing for CMD, and advances in the accuracy and availability of these has now been reflected in contemporary guidelines. The 2021 ACC/AHA Guideline for the Evaluation and Diagnosis of Chest Pain^14^ gives stress PET or stress CMR with MBFR a IIa recommendation, and stress echocardiography with MBVR a IIb recommendation, for the diagnosis of CMD in patients with stable chest pain and suspected INOCA.

## PET

Myocardial perfusion imaging using stress/rest PET is the most well established and validated non-invasive method for the global detection of CMD. As a result, it is currently the most widely used non-invasive method and is recognized as the gold standard reference.^25^ PET uses radiotracers (e.g., ^82^Rb, ^13^N-ammonia, ^15^O-H_2_O) and dynamic first-pass vasodilator stress and then rest imaging to quantify absolute myocardial blood flow (MBF) at stress (maximal hyperemia) and rest in mL·g^−1^·min^−1^. Post-processing software gives accurate measurements for both regional and global stress and rest MBF.^26–28^

These measurements then allow for the calculation of the myocardial perfusion reserve (MPR), defined as the ratio of stress MBF to rest MBF. An MPR < 2.0, in the absence of obstructive CAD, is widely accepted as the diagnostic threshold for CMD. MPR has been shown to be an independent predictor of major cardiovascular events.^29^ In addition, use of MPR improves risk stratification in patients with suspected CMD.^30^

MBF and MPR measured by PET have been validated against invasive measurements in several studies, and have been shown to be accurate and reproducible.^31^ An advantage of PET over other diagnostic methods is that it allows for global assessment of coronary microvascular function in all coronary territories. This is particularly important as it is recognized that CMD often has a heterogeneous distribution over the myocardium, and diagnosis may be missed if only one coronary territory is assessed (e.g., by invasive angiographic assessment of a single coronary artery).^32^ Furthermore, PET involves relatively low radiation exposure. This can be reduced even further by the use of high-sensitivity 3D PET scanners, while still allowing for accurate MBF quantification.^33,34^

PET is widely available in many healthcare systems, notably in North America and some parts of Europe. Elsewhere, as is also the case with CMR, PET is less available in part due to costs and logistics. Further, interpretation of myocardial perfusion scans (PET or CMR) for CMD are facilitated by coronary angiography to clarify the presence, distribution, and severity of CAD before CMD can be diagnosed. PET imaging offers slightly lower spatial resolution than CMR (typical spatial resolution for PET = 5-7 mm, for CMR = 1.5 × 1.5 × 10 mm^3^).^35^

## CMR

Dynamic first-pass imaging of MBF using cardiovascular magnetic resonance (CMR) is well established for the evaluation of suspected obstructive CAD. However, more recently stress perfusion CMR has increasingly been used to assess for CMD.

Historically, this technique involved semi-quantitative, visual interpretation of stress/rest CMR perfusion scans. Dynamic first-pass vasodilator stress imaging is followed by rest perfusion imaging to calculate a semi-quantitative MPR index (MPRI), which correlated with CFR.^36^ This qualitative approach had some diagnostic and prognostic value for patients with CMD in the setting of non-obstructive CAD.^36^ However, this method had inherent limitations in diagnostic accuracy, mainly due to variations in MPRI related to resting flow.

**Figure 2:**
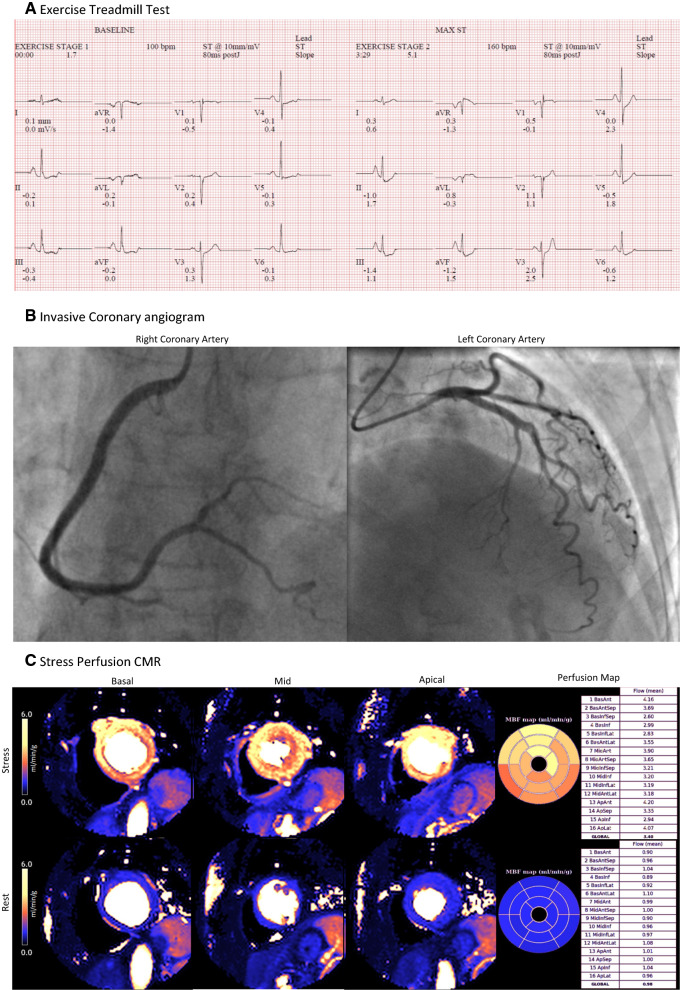
Multi-modality investigations for a 66 year old woman referred to the cardiology clinic with chest pain. **A** Exercise Treadmill Test was inconclusive with minor upsloping ST depression without symptoms. **B** Invasive coronary angiogram showed smooth unobstructed coronary arteries. **C** Stress/rest perfusion CMR at 1.5 T coupled with inline pixel mapping of myocardial blood flow revealed normal myocardial perfusion, with normal stress MBF (Global stress MBF = 3.40 mL·min^−1^·g^−1^) and normal MPR (Global MPR = 3.47). The final diagnosis was non-cardiac chest pain (Acknowledgement Dr. P. Kellman and Dr. H Xue, National Institutes of Health)

**Figure 3:**
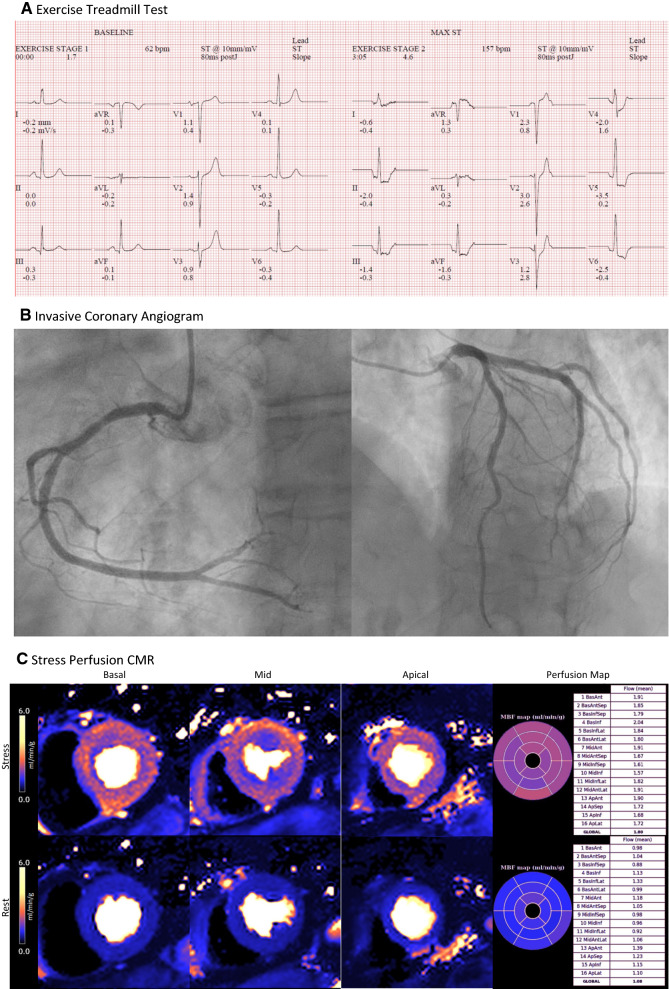
Multi-modality investigations in a 70 year old woman with recurrent hospitalizations with chest pain and consistently associated with high-sensitivity troponin I concentrations within the normal sex-specific range (< 16 ng·L^−1^). **A** The exercise treadmill test was strongly positive for ischemia with widespread horizontal ST depression. **B** The invasive coronary angiogram disclosed minor atherosclerotic plaque only, with no obstructive CAD. **C** Stress/rest perfusion CMR imaging coupled with inline pixel mapping of myocardial blood flow revealed a circumferential subendocardial perfusion defect, low stress MBF (Global stress MBF = 1.80 mL·min^−1^·g^−1^), and low MPR (Global MPR = 1.67). These findings are diagnostic of CMD. The final diagnosis was microvascular angina (Acknowledgement Dr. P. Kellman and Dr. H Xue, National Institutes of Health)

Recently, there have been significant advances and the development of fully automatic, pixel-wise quantitative mapping of myocardial perfusion by CMR (Figures 2 and 3). This method developed by Kellman and colleagues automatically generates pixel-encoded maps of MBF (mL·min^−1^·g^−1^ tissue) which are acquired during vasodilator (hyperemic) stress and resting conditions. MPR may therefore be calculated in a similar way to PET.^37–39^ This method has been validated against both invasive measures of coronary function and PET.^38,40^ Furthermore, it has been shown that MBF and MPR measured using this method are strong independent predictors of adverse cardiovascular outcomes.^41^

MBF may be considered at a global level (all segments) or at a segmental level (16- or 32-segments) and prior studies have established a cut-off for global hyperemic blood flow of 2.25 mL·min^−1^·g^−1^ tissue.^42^ Since the microcirculation has a sub-endocardial distribution, microvascular dysfunction may be spatially localized with a ‘sub-segmental’ distribution of hypoperfusion relative to the sub-epicardium. This threshold of 2.25 mL·min^−1^·g^−1^ may be applied to sub-endocardial segments.^42^ Myocardial perfusion ratio (MPR) represents the ratio of hyperemic blood flow achieved during stress testing indexed to resting blood flow. MPR can also be calculated specifically for the subendocardial layer (MPR_ENDO_) (defined as: hyperemic MBP_ENDO_/rest MBF_ENDO_). A MPR_ENDO_ of 2.41 has been identified a specific cut-off for microvascular dysfunction.^43^ The average resting blood flow is approximately 0.6-0.8 mL·min^−1^·g^−1^ tissue, however, resting blood flow may vary within the adult population, and this is especially the case in women^43,44^ and in patients with diabetes^45^ who commonly have ‘high’ resting blood flow > 1.0 mL·min^−1^·g^−1^ tissue. In these patients, MPR may be falsely low. An MPR threshold of 2.2 has been proposed as a cut-off for microvascular dysfunction.^43^

Other methods using stress CMR to detect CMD are also being developed. Adenosine stress CMR T1-mapping has potential for detection of microvascular dysfunction in patients with type 2 diabetes without obstructive CAD^[46^ and may, potentially, distinguish epicardial from microvascular coronary disease.^47^ This method has the added advantage of not requiring gadolinium contrast media, and therefore would be suitable for patient with severe renal impairment, in whom stress perfusion CMR is usually contraindicated.

A major advantage of CMR is that it allows for assessment of cardiac structure, function, and tissue characterization of myocardial scar, inflammation and extracellular volume, while simultaneously assessing for CMD. There is no exposure to ionizing radiation, and all coronary artery territories are simultaneously assessed, as is the case with PET. However, CMR does have some limitations, notably comparatively high costs and ineligible patient groups, e.g., severe renal disease, claustrophobia, implantable devices. And while CMR is generally more accessible than PET, software for quantification of MBF is not universally available.

## Cardiac CT

CT coronary angiography is well established for the assessment anatomical assessment of CAD. However, there have been advances in the use of CT for functional assessment as well. Myocardial first-pass dynamic CT allows for semi-quantitative assessment of MBF and MPR.^48,49^ However, there have been very few studies investigating its ability to detect CMD and it has not been validated. The main advantage of CT would be the ability to combine anatomical and functional imaging in one imaging examination, reducing the need for additional investigations. However, CT involves exposure to ionizing radiation and the risk of contrast-induced nephropathy. Also, iodine-based contrast media risks overestimation of MBF, due to the vasodilatory properties of the contrast.

## Echocardiography

Transthoracic Doppler echocardiography (TTDE) of the left anterior descending (LAD) coronary artery can be used to calculate the coronary flow velocity ratio (CFVR). This is calculated by the ratio of peak diastolic flow in the artery at rest and at stress (induced by adenosine, dipyridamole, or regadenoson).^50^ In the absence of obstructive epicardial CAD, CFVR acts as a measure of coronary microvascular function. There is a lack of consensus on an exact threshold, but CFRV ≤ 2.0-2.5 is thought to be consistent with CMD.^13^

TTDE is comparatively inexpensive compared with other imaging modalities, is readily available, and involves no radiation exposure. However, limitations include operator dependency, and the need for extensive training. There will be patient related technical issues that are encountered with all forms of transthoracic echo (e.g., poor acoustic windows). Furthermore, TTDE is mainly limited to assessing the LAD territory, and therefore fails to account for heterogeneous microvascular dysfunction. Myocardial contrast echocardiography using intravenous injection of microbubbles to assess MPR has been validated against PET,^51^ however, it is rarely used in clinical practice.

### Single-Photon Emission Computed Tomography

Single-photon emission computed tomography (SPECT) uses gamma rays for measuring MBF and MFR. Until recently, the use of SPECT for detection of CMD was largely limited by the pharmacokinetics of the radiotracers used. However, advances in SPECT with cadmium zinc telluride (CZT) detectors now allows quantification of MBF and MFR, and studies have shown reasonable correlation between PET and CZT SPECT.^52^

## Epidemiology

### Prevalence

CMD may occur in the presence or absence of CAD and/or myocardial disease (Table 1). Most patients referred for a noninvasive computed tomography coronary angiogram (CTA) or invasive coronary angiogram for the investigation of angina do not have obstructive CAD.^4^ Many of these patients may have underlying CMD. However, establishing the prevalence with reliable accuracy and precision has been challenging. Reasons include the lack of specific noninvasive testing, methodological differences between diagnostic methods, variations in the diagnostic threshold used to define CMD, selected populations, and sample size.

One of the largest studies examining the prevalence of CMD in patients with chest pain and non-obstructive CAD used invasive coronary function testing (including ACh reactivity testing) to diagnose CMD in 1,439 patients. They found that more than two-thirds of patients had some form of CMD (1,171/1,439).^7^ These findings were in keeping with a previous smaller study in which patients with angina and non-obstructive CAD were assessed in the same way. This study found that 59% (120/203) patients had CMD.^53^

The Women’s Ischemia Syndrome Evaluation (WISE) Study found that 74/189 (39%) of women with chest pain but normal coronary arteries had an abnormal CFR consistent with CMD.^54^ Similar results have been demonstrated using non-invasive diagnostic methods. In a large study of 1,218 patients with angina but no history of CAD and no visual evidence of CAD on rest/stress PET myocardial perfusion imaging, PET was used to quantify CFR to investigate for the presence of CMD (CFR < 2.0 = abnormal). They found that CMD was highly prevalent with 641/1,218 (53%) of patients having CFR < 2.0, consistent with CMD.^55^

However, a lower prevalence was noted in the iPOWER study in which 241/963 (26%) with chest pain but no obstructive CAD on angiography, had markedly impaired CFRV consistent with CMD when measured by transthoracic Doppler echo.^56^ This finding could be related to the use of echocardiography (and related reduced test sensitivity for CMD).

A recent systematic review investigating the prevalence of microvascular angina in patients with stable angina in the absence of obstructive CAD, defined MVA in different settings according to the COVADIS criteria. They found a median prevalence of 43% for suspected MVA using non-invasive ischemia testing, 28% for suspected MVA using specific modalities for MVA, and 30% for definite MVA.^57^

The Coronary Microvascular Function and CT Coronary Angiogram (CorCTCA) study is a multicenter cohort study and stratified medicine trial that will prospectively characterize the prevalence of disease endotypes in 250 patients with known or suspected angina and no obstructive coronary arteries,^58^ as defined by coronary CTA. The nested randomized, controlled trial will assess whether disclosure of the endotypes with linked therapy will improve health and economic outcomes, as compared to standard coronary CTA guided management.

### Sex

Most individuals who have anginal symptoms and no obstructive coronary arteries are female.^4,8^ Despite the lower prevalence of obstructive CAD, women have a similar prevalence of ST elevation myocardial infarction, and have excess mortality compared with men.^59^ CMD is not a benign condition; it associated with increased morbidity and major adverse cardiovascular events,^8,60^ and CMD is likely to contribute to the cardiovascular morbidity encountered by women with INOCA.

Meta-analyses have shown that CMD is more prevalent in women,^57^ and particularly among post-menopausal women.^54^ Other large studies have shown that while the prevalence of CMD is higher in women than men, the prevalence was generally high in both populations and is associated with adverse outcomes regardless of sex.^53,55^

### Traditional Cardiovascular Risk Factors

Multiple cardiovascular risk factors are associated with increased prevalence of CMD. Several large studies, including the WISE, iPOWER, and CorMicA studies, have shown that hypertension, dyslipidemia, diabetes, and age are all associated with impaired coronary microvascular function.^54,56,61^ CMD is also more prevalent in inflammatory conditions,^62–64^ and has been shown to be associated with serum biomarkers of inflammation.^65,66^

## Future Developments

CMD is highly prevalent and has significant unmet clinical need. More research and large-scale studies are therefore urgently needed to address this important health condition.

Encouragingly there are multiple current studies of the prevalence, detection, and treatment of CMD.

Multiple non-invasive methods for the detection of CMD are now available, but there is a paucity of evidence that management guided by noninvasive imaging improves clinical outcomes The CorCMR trial (NCT04805814) will assess the clinical utility of quantitative stress perfusion CMR for the diagnosis of CMD and determine whether stratified medical therapy guided by the results of the stress perfusion CMR improves symptoms, well-being, cardiovascular risk and health and economic outcomes.

At present there are no evidence-based, disease-modifying therapies for the treatment of CMD. Some studies have suggested that intensive medical therapy including statins and angiotensin converting enzyme inhibitors (ACE-I) or receptor blockers (ARB) may be beneficial. The Women’s Ischemia Trial to Reduce Events In Non-Obstructive CAD (WARRIOR) trial (NCT03417388) is a multicenter, prospective, randomized, blinded outcome evaluation, to assess the impact of intensive medical therapy (high dose statin + ACEi/ARB) versus usual care on major adverse cardiovascular events in women with INOCA.^67^

There is also much anticipation for the Precision Medicine with Zibotentan in Microvascular Angina (PRIZE) trial (NCT04097314). Zibotentan is a potent, oral, selective inhibitor of the endothelin A receptor. Dysregulation of the endothelin system is implicated in the development of CMD, and therefore zibotentan has potential as a disease-modifying therapy for CMD. The PRIZE trial will assess the efficacy and safety of zibotentan as a novel treatment for CMD.^68^

## Conclusion

CMD is a highly prevalent and important health problem, however, it often goes unrecognized by clinicians. Accurate diagnosis and stratified medical therapy are crucial to improve symptoms, quality of life, and long-term outcomes for patients with CMD. International standardized criteria provide a structured approach for the diagnosis of this condition, and the use of both invasive and non-invasive diagnostic methods are essential for the accurate diagnosis of CMD. However, ongoing research is required to continue to develop diagnostic methods and novel disease-modifying therapies.

## Supplementary Information

Below is the link to the electronic supplementary material.

Supplementary file1 (PPTX 3639 kb)

## Abbreviations

CAD: Coronary artery disease
CFR: Coronary flow reserve
CMD: Coronary microvascular disease
CMR: Cardiovascular magnetic resonance
COVADIS: COVADIS: Coronary Vasomotion Disorders International Study Group
IHDT: Ischemic heart disease
IMR: Index of microcirculatory resistance
MBF: Myocardial blood flow
MPR: Myocardial perfusion reserve
PET: Positron emission tomography
