# Supplementary material for: Definition and epidemiology of coronary microvascular disease
Source: J Nucl Cardiol. 2022 May 9;29(4):1763–75. doi: 10.1007/s12350-022-02974-x (PMC9345825; doi:10.1007/s12350-022-02974-x)
Supplement: Supplementary file 1 — Supplementary file1 (PPTX 3639 kb) [file 12350_2022_2974_MOESM1_ESM.pptx]

## Slide 1
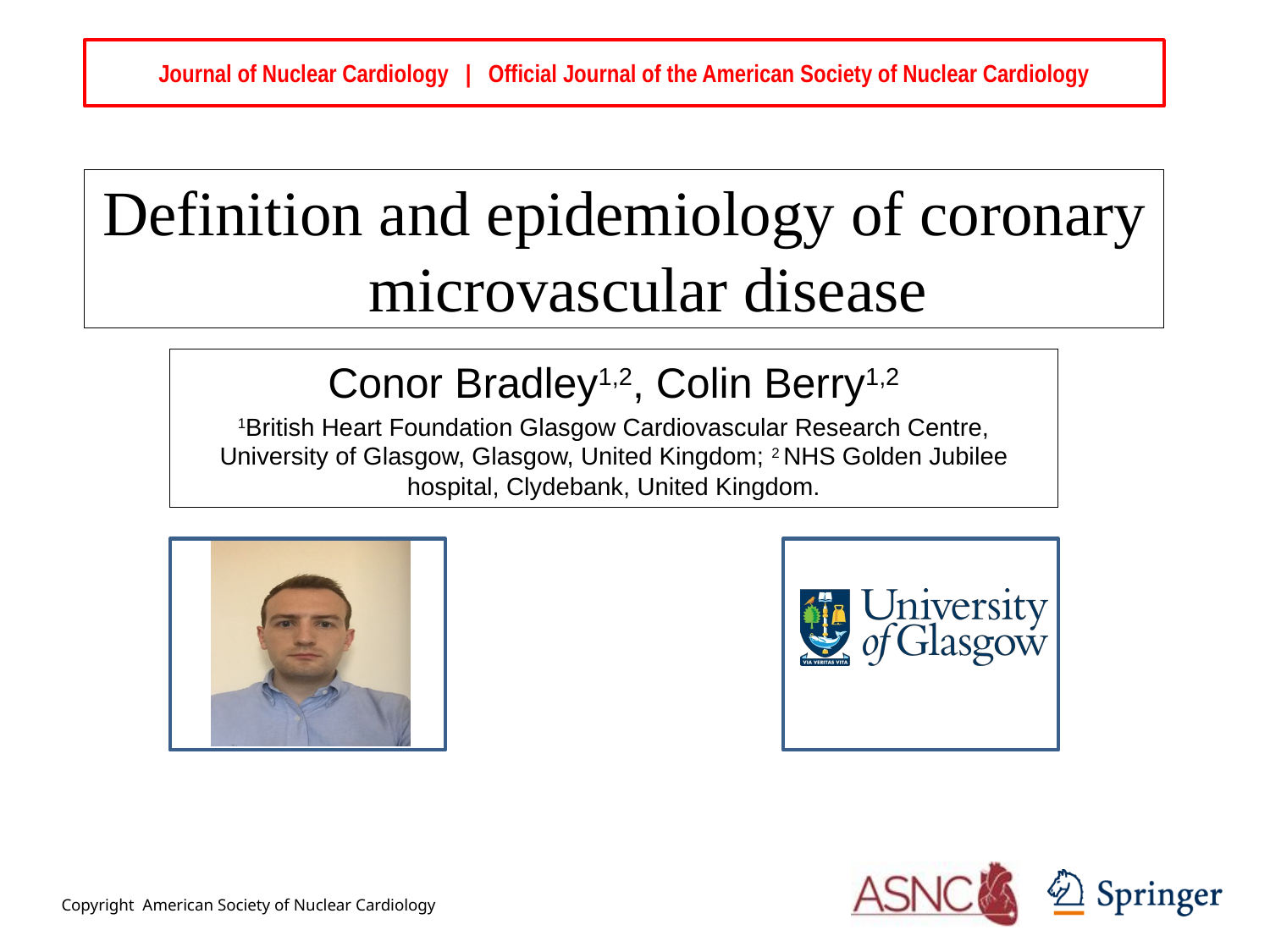

Journal of Nuclear Cardiology | Official Journal of the American Society of Nuclear Cardiology
# Definition and epidemiology of coronary microvascular disease
Conor Bradley1,2, Colin Berry1,2
1British Heart Foundation Glasgow Cardiovascular Research Centre, University of Glasgow, Glasgow, United Kingdom; 2 NHS Golden Jubilee hospital, Clydebank, United Kingdom.
Head shot of author
required
Copyright American Society of Nuclear Cardiology

## Slide 2
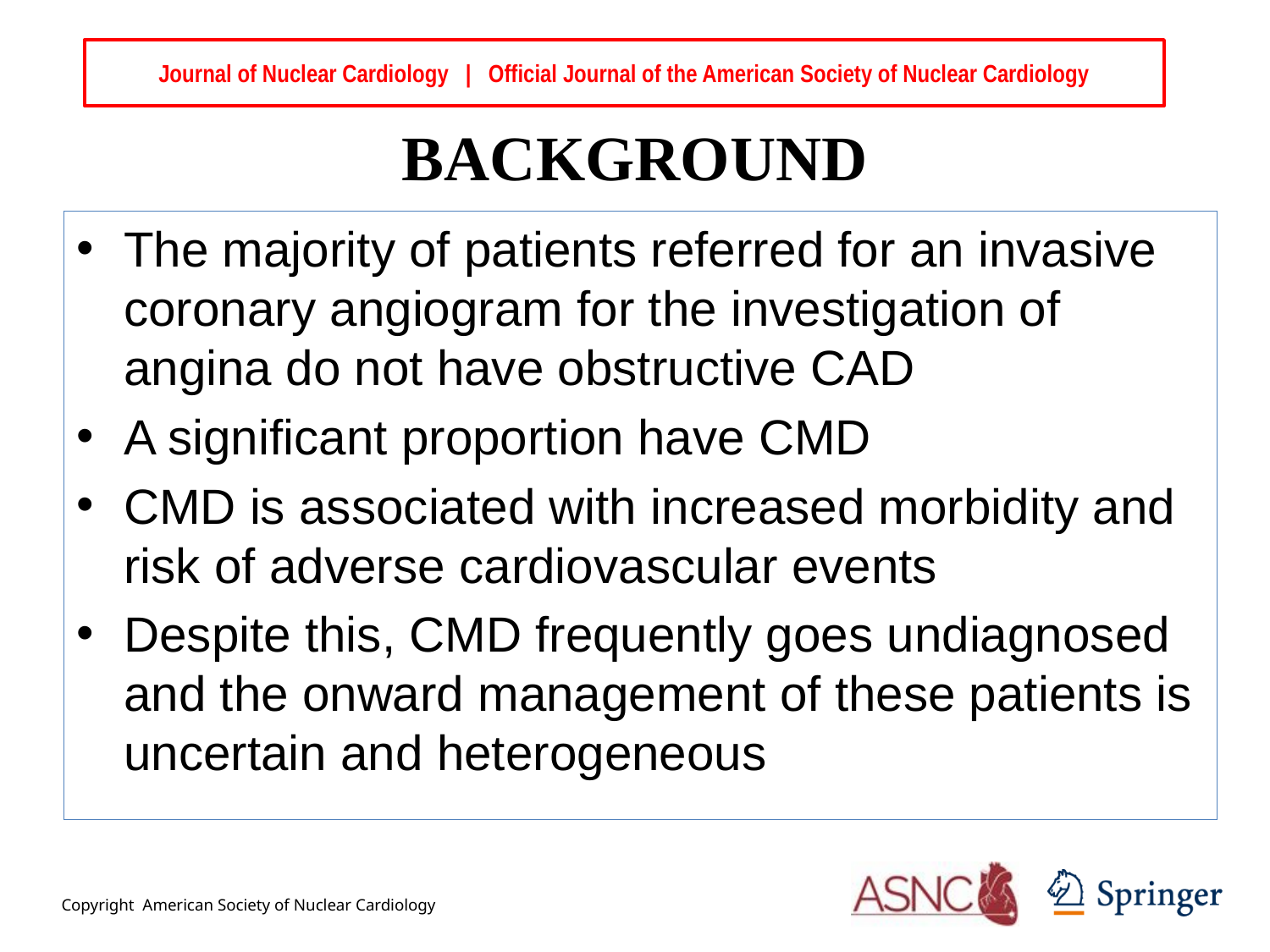

Journal of Nuclear Cardiology | Official Journal of the American Society of Nuclear Cardiology
# BACKGROUND
The majority of patients referred for an invasive coronary angiogram for the investigation of angina do not have obstructive CAD
A significant proportion have CMD
CMD is associated with increased morbidity and risk of adverse cardiovascular events
Despite this, CMD frequently goes undiagnosed and the onward management of these patients is uncertain and heterogeneous
Copyright American Society of Nuclear Cardiology

## Slide 3
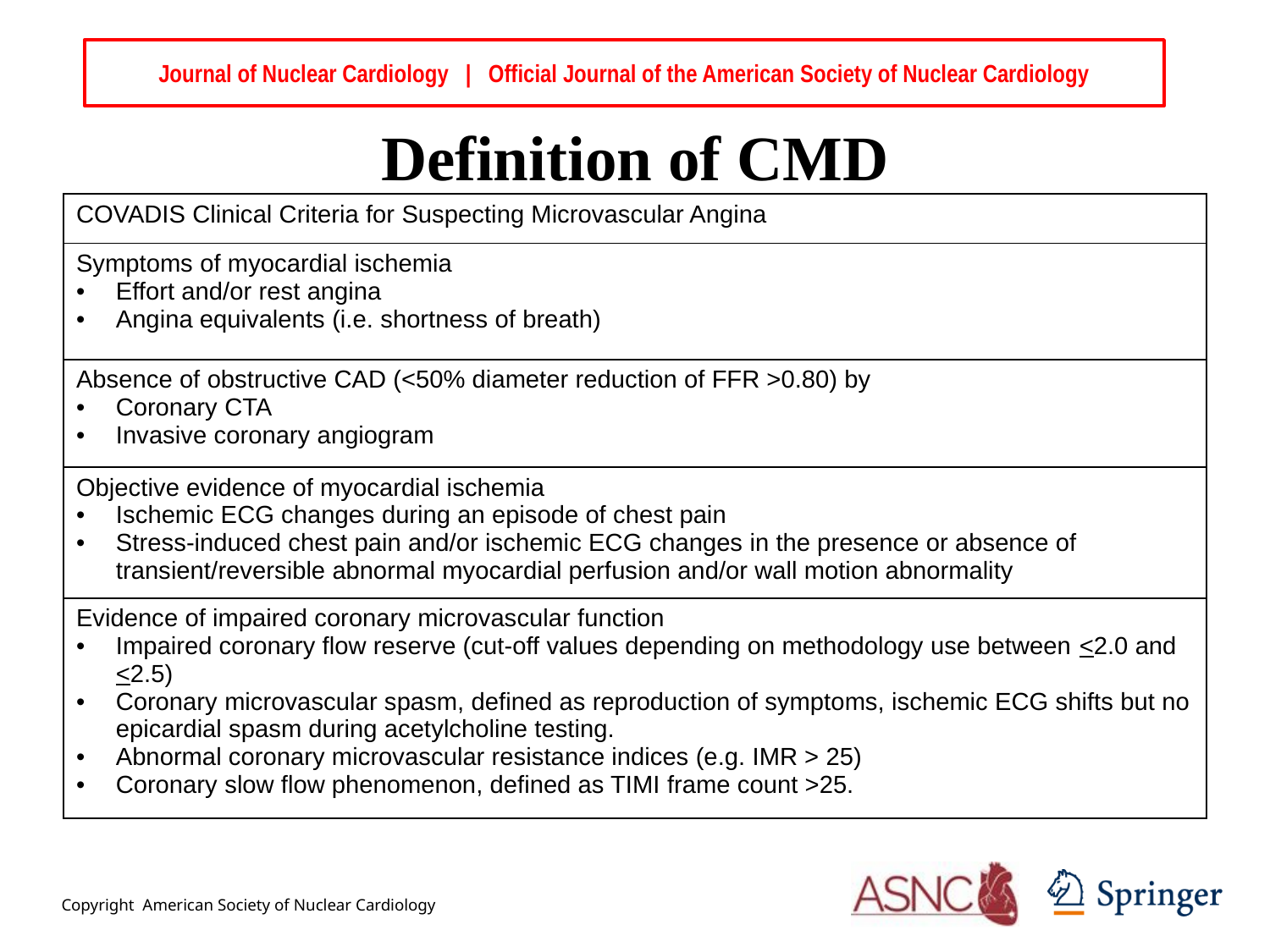

Journal of Nuclear Cardiology | Official Journal of the American Society of Nuclear Cardiology
# Definition of CMD
| COVADIS Clinical Criteria for Suspecting Microvascular Angina |
| --- |
| Symptoms of myocardial ischemia Effort and/or rest angina Angina equivalents (i.e. shortness of breath) |
| Absence of obstructive CAD (<50% diameter reduction of FFR >0.80) by Coronary CTA Invasive coronary angiogram |
| Objective evidence of myocardial ischemia Ischemic ECG changes during an episode of chest pain Stress-induced chest pain and/or ischemic ECG changes in the presence or absence of transient/reversible abnormal myocardial perfusion and/or wall motion abnormality |
| Evidence of impaired coronary microvascular function Impaired coronary flow reserve (cut-off values depending on methodology use between <2.0 and <2.5) Coronary microvascular spasm, defined as reproduction of symptoms, ischemic ECG shifts but no epicardial spasm during acetylcholine testing. Abnormal coronary microvascular resistance indices (e.g. IMR > 25) Coronary slow flow phenomenon, defined as TIMI frame count >25. |
Copyright American Society of Nuclear Cardiology

## Slide 4
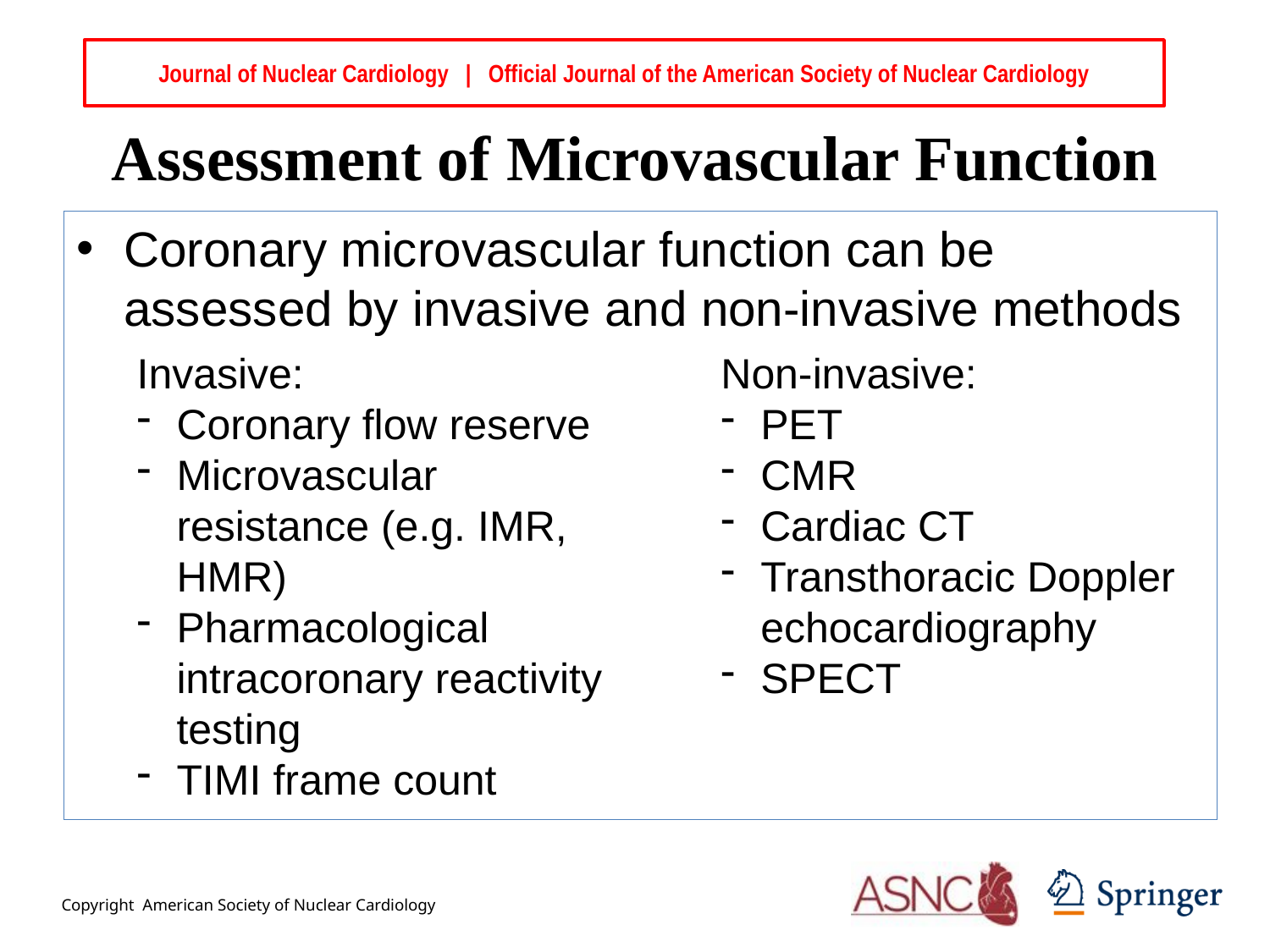

Journal of Nuclear Cardiology | Official Journal of the American Society of Nuclear Cardiology
# Assessment of Microvascular Function
Coronary microvascular function can be assessed by invasive and non-invasive methods
Invasive:
Coronary flow reserve
Microvascular resistance (e.g. IMR, HMR)
Pharmacological intracoronary reactivity testing
TIMI frame count
Non-invasive:
PET
CMR
Cardiac CT
Transthoracic Doppler echocardiography
SPECT
Copyright American Society of Nuclear Cardiology

## Slide 5
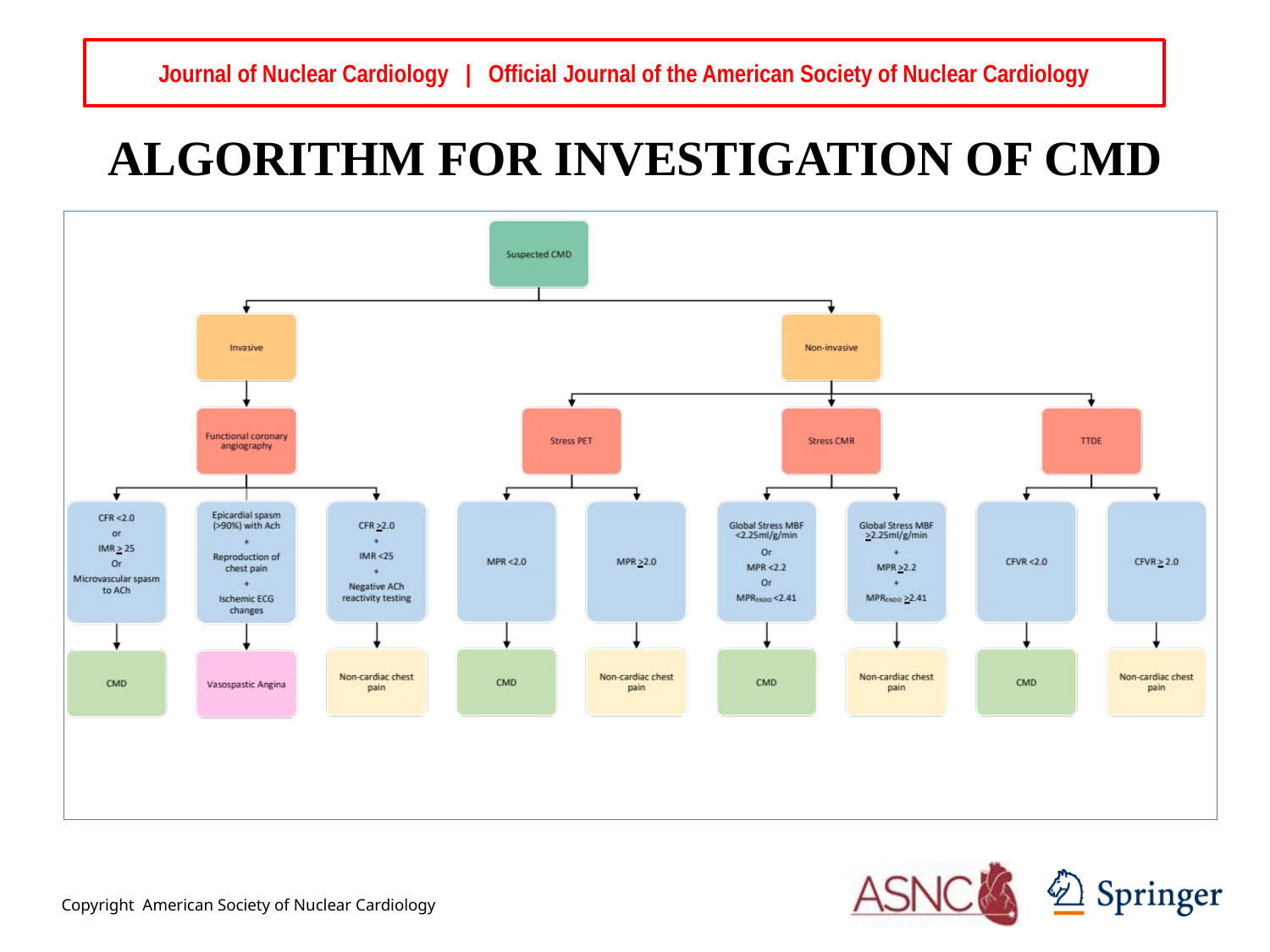

Journal of Nuclear Cardiology | Official Journal of the American Society of Nuclear Cardiology
# ALGORITHM FOR INVESTIGATION OF CMD
Copyright American Society of Nuclear Cardiology

## Slide 6
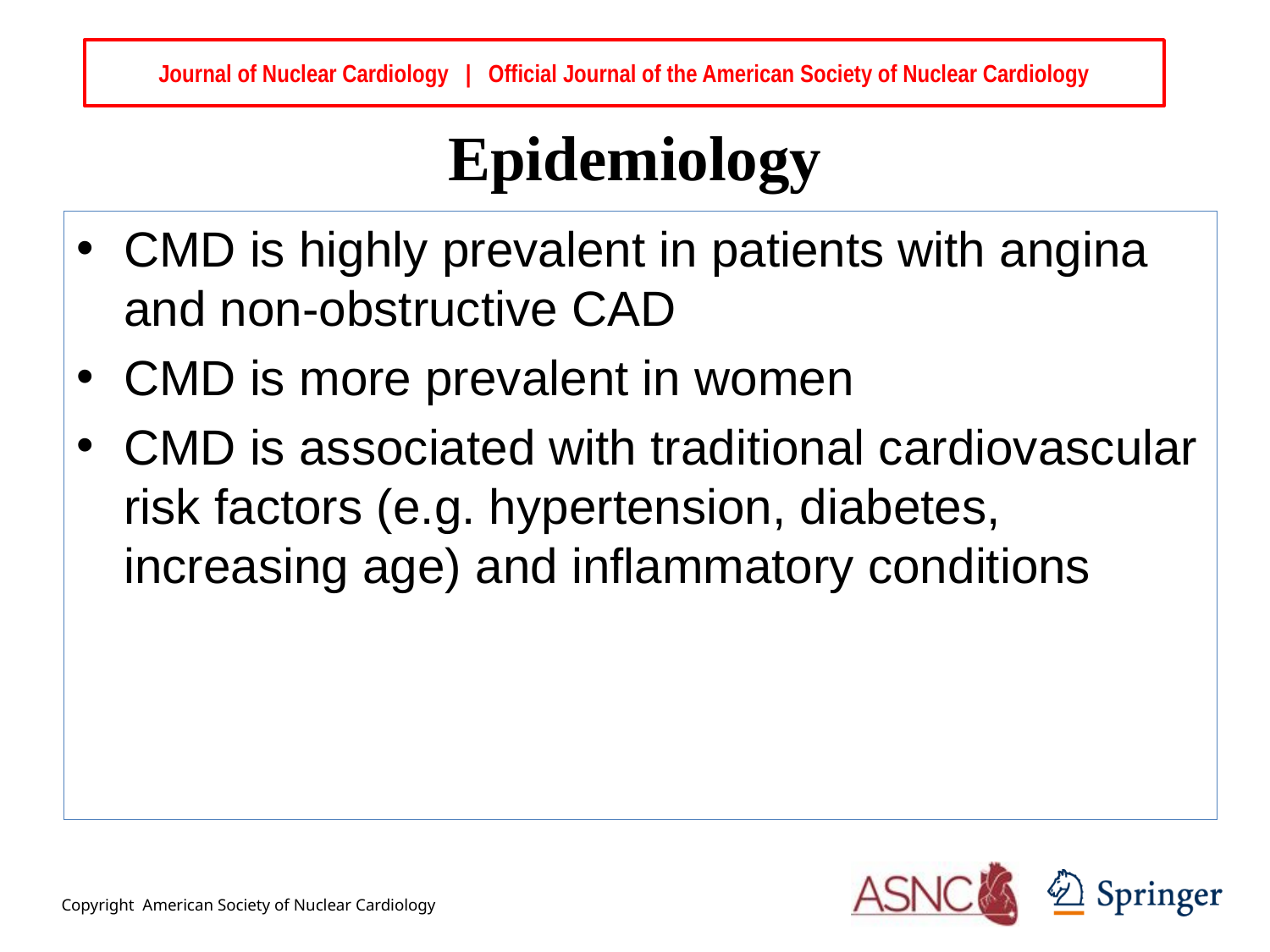

Journal of Nuclear Cardiology | Official Journal of the American Society of Nuclear Cardiology
# Epidemiology
CMD is highly prevalent in patients with angina and non-obstructive CAD
CMD is more prevalent in women
CMD is associated with traditional cardiovascular risk factors (e.g. hypertension, diabetes, increasing age) and inflammatory conditions
Copyright American Society of Nuclear Cardiology

## Slide 7
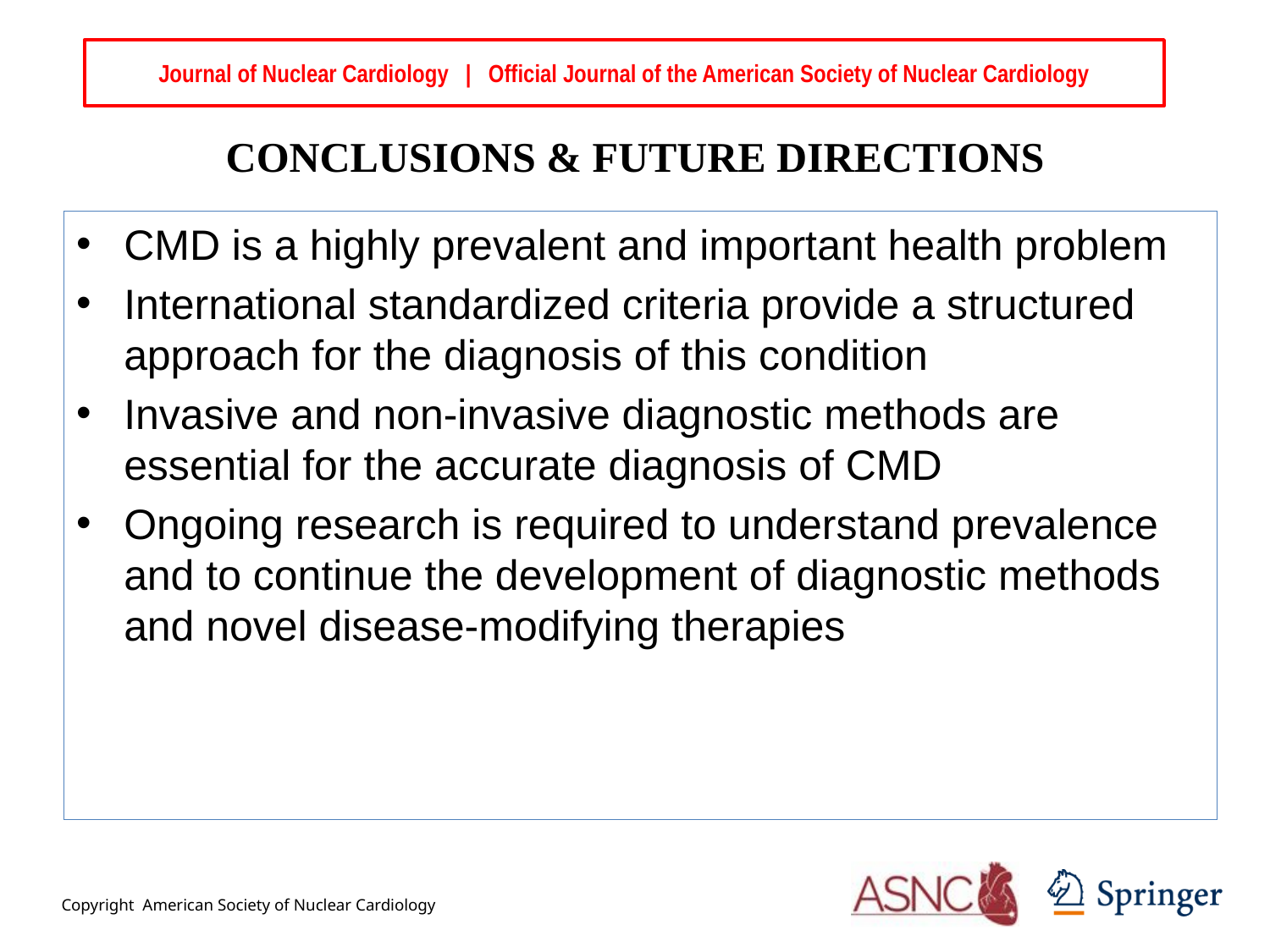

Journal of Nuclear Cardiology | Official Journal of the American Society of Nuclear Cardiology
# CONCLUSIONS & FUTURE DIRECTIONS
CMD is a highly prevalent and important health problem
International standardized criteria provide a structured approach for the diagnosis of this condition
Invasive and non-invasive diagnostic methods are essential for the accurate diagnosis of CMD
Ongoing research is required to understand prevalence and to continue the development of diagnostic methods and novel disease-modifying therapies
Copyright American Society of Nuclear Cardiology
